# The Role of Nutrition and Nutritional Supplements in Ocular Surface Diseases

**DOI:** 10.3390/nu12040952

**Published:** 2020-03-30

**Authors:** Marco Pellegrini, Carlotta Senni, Federico Bernabei, Arrigo F. G. Cicero, Aldo Vagge, Antonio Maestri, Vincenzo Scorcia, Giuseppe Giannaccare

**Affiliations:** 1 Ophthalmology Unit, S.Orsola-Malpighi University Hospital, University of Bologna, 40138 Bologna, Italy; c.senni3@gmail.com (C.S.); federico.bernabei89@gmail.com (F.B.); 2 Medical and Surgical Sciences Department, University of Bologna, 40138 Bologna, Italy; arrigo.cicero@unibo.it; 3 Eye Clinic of Genoa, Policlinico San Martino, Department of Neuroscience, Rehabilitation, Ophthalmology, Genetics, Maternal and Child Health (DiNOGMI), University of Genoa, 16132 Genoa, Italy; aldo.vagge@gmail.com; 4 Medical Oncology Department, Santa Maria della Scaletta Hospital, 40026 Imola, Italy; a.maestri@ausl.imola.bo.it; 5 Department of Ophthalmology, University Magna Græcia of Catanzaro, 88100 Catanzaro, Italy; vscorcia@libero.it (V.S.); giuseppe.giannaccare@gmail.com (G.G.)

**Keywords:** ocular surface, dry eye disease, nutritional supplements, nutraceuticals

## Abstract

Dry eye disease (DED) is a multifactorial disease of the ocular surface system whose chore mechanisms are tear film instability, inflammation, tear hyperosmolarity and epithelial damage. In recent years, novel therapies specifically targeting inflammation and oxidative stress are being investigated and used in this field. Therefore, an increasing body of evidence supporting the possible role of different micronutrients and nutraceutical products for the treatment of ocular surface diseases is now available. In the present review, we analyzed in detail the effects on ocular surface of omega-3 fatty acids, vitamins A, B12, C, D, selenium, curcumin and flavonoids. Among these, the efficacy of omega-3 fatty acid supplementation in ameliorating DED signs and symptoms is supported by robust scientific evidence. Further long-term clinical trials are warranted to confirm the safety and efficacy of the supplementation of the other micronutrients and nutraceuticals.

## 1. Introduction

### 1.1. The Ocular Surface System

The ocular surface system consists of various components of the eye that are structurally and functionally linked by continuous epithelia and common nervous, endocrine, vascular and immune systems. It includes cornea, conjunctiva, lacrimal and meibomian glands, nasolacrimal duct and eyelids [1]. Being a direct connection between the eye and the external environment, all components act synergistically to maintain a healthy and well-performing refractive status and to protect the key structures of vision against external pathogenic noxa. Both the aforementioned functions of the system are ensured by the production of an efficient tear film. In particular, lacrimal glands secrete the watery component as well as electrolytes and protective proteins; the corneal and conjunctival epithelia release mucins that transform the aqueous tears into a muco-aqueous gel and increase lubrication; meibomian glands secrete lipids (meibum) that form the outermost layer of the tear film, preventing its evaporation. Any factor perturbing the homeostasis of the ocular surface system may disrupt the stability and osmolarity of the tears, leading to tissue damage by means of osmotic, mechanical and inflammatory mechanisms [2].

### 1.2. Dry Eye Disease

Dry eye disease (DED) is a multifactorial disease of the ocular surface system that affects 5% to 30% of individuals over the age of 50 [3]. DED has a higher incidence in older people, postmenopausal women, contact lens wearers and patients with autoimmune conditions. Symptoms of DED, such as blurry vision, light sensitivity, irritation, burning and itching, may restrict daily activities with negative impact on quality of life [4].

**Figure 1:**
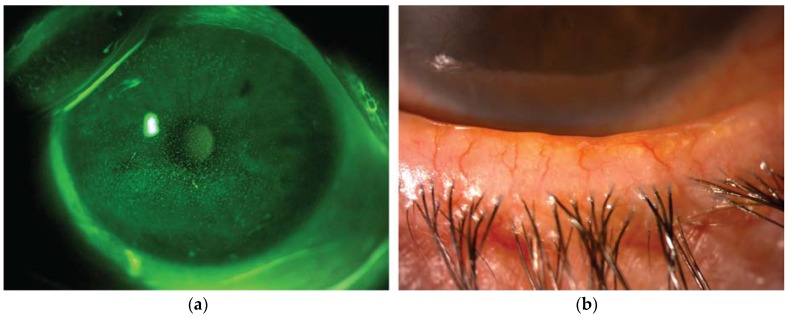
Representative image from a patient with dry eye disease owing to Meibomian gland dysfunction. (**a**) Slit lamp photograph of the cornea after instillation of 20 µL unpreserved 2% sodium fluorescein (corneal fluorescein staining). The epithelial damage is visible as multiple punctate epithelial erosions staining with fluorescein scattered over the corneal surface. (**b**) Slit lamp photograph of the lid margin showing typical signs of meibomian gland dysfunction, including hyperemia and irregularity of the lid margin, telangectasia, plugged gland orifices and anterior displacement of the mucocutaneous junction. Hyposecretion of meibomian lipids leads to tear film instability and increased evaporation rate.

A combination of subjective questionnaires and diagnostic tests are used to diagnose DED and score its severity. The stability of the tear film is estimated by measuring the time interval between a complete blink and the appearance of the first break in the tear film (break-up time). The tear volume is evaluated by measuring the length of wetting of paper strips kept into the temporal lower conjunctival sac for 5 minutes (Schirmer test), or more recently by assessing the height of the inferior tear film meniscus by means of anterior segment optical coherence tomography. The measurement of tear osmolarity is considered one of the best metric to grade the DED severity [5]. Finally, dyes such as fluorescein, rose bengal, and lissamine green are used to stain the ocular surface and evaluate the epithelial integrity (Figure 1a) [6].

The classification of DED includes two main sub-types that are characterized by lower production of tear (aqueous deficient DED) or increased evaporation (evaporative DED), which often coexist in the same clinical picture. The latter form is typically associated with meibomian gland dysfunction, a quali/quantitative alteration of the lipids produced by these glands, which are essential to prevent excessive evaporation of the tear film (Figure 1b) [7]. Both low production and high evaporation of tears lead to hyperosmolarity and subsequent inflammation, which cause epithelial damage and loss of goblet cells. The resulting tear film instability amplifies tear hyperosmolarity, thus self-maintaining the vicious circle of DED [8,9].

### 1.3. The Role of Oxidative Stress and Inflammation

The ocular surface is almost constantly exposed to sunlight, which consists of wavelengths including ultraviolet light, a well-recognized causative factor of oxidative stress. Under normal conditions, antioxidant enzymes, such as superoxide dismutase, catalase and glutathione peroxidase, eliminate the reactive oxygen species generated by oxidative events. However, several factors can lead to the perturbation of this delicate balance. For instance, inflammatory cytokines such as interleukin (IL)-1β, IL-6, IL-8, and tumor necrosis factor (TNF)-α, which are up-regulated in many ocular surface diseases, increase the expression of reactive nitrogen species. Not surprisingly, oxidative stress has an important pathogenetic role in the setting of Sjögren syndrome, a systemic autoimmune disease that involves the lacrimal causing severe DED, as suggested by the association between the generation of reactive oxygen species and the lipid peroxidation, leading to damage to the cellular membrane of ocular surface epithelia [10]. Furthermore, ageing is an established risk factor for oxidative stress, as the concentration of endogenous antioxidant species significantly decreases with increasing age, while reactive oxygen species production increases [11]. Indeed, physiological processes progressively lose their efficacy and, as a result of such condition, the senescent cell up-regulates the expression of genes encoding a variety of proteins including inflammatory mediators and matrix-remodeling factors, which disrupt the local homeostasis, leading to the creation and maintenance of oxidative stress [12].

Dry eye disease has a strong association with oxidative stress. This is supported by the increased expression of oxidative products and the concomitant decrease of antioxidant agents detected in DED patients. Nakamura and co-authors demonstrated the role of oxidative stress in the pathophysiology of DED. They adopted an experimental model of keratopathy in which oxidative stress was assessed by detecting the levels of damaged DNA and altered proteins by means of immunochemistry. Significant increase of oxidative stress markers and reactive oxygen species were detected in this DED model. Moreover, the disturbance of epithelial differentiation capacity characterized by decreased proliferation, upward migration and increased apoptotic cells, was found to be present in this model, suggesting that chronic exposure to an oxidative environment activates cell regulatory molecules, such as tumor suppressor protein p53, which in turn alters the regenerative capacity of the corneal epithelia and further stimulate the inflammatory cascade [13].

### 1.4. Treatment Strategies

The first line treatment of DED consists of artificial tears to nourish the ocular surface, increase lubrication while concomitantly diluting the concentration of inflammatory cytokines. Topical corticosteroids are effective to control ocular surface inflammation, but their use is associated with potentially serious adverse effects, such as glaucoma, cataract, increased risk of infection and delayed wound healing. Cyclosporine A has a better safety profile but its efficacy is lower, particularly in the short-term period, and its availability on the market (particularly in Europe) is currently limited [14]. Other possible strategies include, among others, punctual occlusion [15], lifitegrast ophthalmic solution [16], eyelid hygiene [17], intense pulsed light treatment [18] and blood-derived eye drops [19,20,21].

Anti-inflammatory and anti-oxidative therapies are considered as causative therapeutic approaches for DED, since they aim at interrupting the underlying vicious circle, rather than providing just a symptomatic temporary relief. In recent years, there has been growing interest in the potential role of nutraceuticals in the prevention and treatment of DED. Numerous in vitro and in vivo studies have demonstrated the beneficial effect of certain dietary constituents on the ocular surface system health [22,23,24,25,26]. The present review will provide a general perspective on the role of nutrition and nutritional supplements in the treatment of ocular surface diseases.

## 2. Essential Fatty Acids 

Omega-3 polyunsaturated fatty acids (FAs) are fundamental structural components of cell membranes as well as precursors for the synthesis of numerous biologically active substances. The main omega-3 FAs include short chain alpha-linoleic acid (ALA), and long-chain eicosapentaenoic acid (EPA), docosapentaenoic acid (DPA) and docosahexanoic acid (DHA). While short-chain omega-3 FAs are obtained from plant sources, long-chain omega-3 FAs are obtained from oily fish and may be synthetized by elongation of short-chain FAs. The biological activity of polyunsaturated FAs depends also on the omega-6 and omega-3 intake ratio. Western diets are associated with an overconsumption of omega-6 FAs, being the ratio typically 15:1, versus an ideal ratio of 4:1 [27].

Omega-3 FAs present anti-inflammatory, anticoagulant and antihypertensive properties, and regulate lipid metabolism, glucose tolerance and central nervous system functions. These molecules exert their anti-inflammatory role by competitive inhibition with arachidonic acid as substrates for the enzymes cyclooxygenase and 5-lypoxygenase [28]. In humans, polyunsaturated FAs have demonstrated a protective effect against chronic diseases such as heart disease, cancer and neurodegenerative disorders [29,30,31].

Recent studies that provided an insight into intrinsic mechanisms of inflammation resolution have pointed out the role of resolvins and protectins derived from omega-3 FAs, which hinder leukocyte infiltration and enhance the clean-up function of macrophages [32]. Resolvins demonstrated anti-inflammatory activity on human corneal epithelial cells in vitro [33], and showed to reduce inflammation, promote corneal epithelial integrity and tear production in a dry eye murine model [34]. Furthermore, corneal lipooxygenases synthetize neuroprotectin D1, a DHA-derived lipid mediator with anti-inflammatory, epitheliotrophic and neuroprotective activities [35].

The possible neuroprotective effect of omega-3 FAs is of great clinical interest for ophthalmologists [36]. In fact, corneal nerves are essential for tear production, protective blink reflex and release of trophic neuromodulators that maintain the vitality and metabolism of ocular surface tissues. Damage to corneal nerve plexus results in a degenerative corneal disease known as neurotrophic keratitis, characterized by spontaneous epithelial breakdown, impaired wound healing and corneal ulceration [37]. In addition, there is increasing evidence in support of a role of neurosensory abnormalities in the etiology of DED [38,39]. DHA potentiates the effect of nerve growth factor (NGF) in stimulating nerve regeneration and epithelial proliferation in rabbit models of corneal neuropathy [40]. Furthermore, in a recent clinical trial, 3 months of omega-3 FAs supplementation resulted in increased nerve corneal nerve fiber length and branch density [41].

The effect of omega-3 FAs supplementation may vary according to DED subtypes. For instance, in patients with meibomian gland dysfunction, their efficacy may depend not only on the anti-inflammatory activity, but also on the effect on the meibomian lipids composition. In mice, an omega-3 FAs deficient diet results in decreased meibum secretion [42]. In cultivated human meibomian gland epithelial cells, exposure to omega-3 and omega-6 FAs influences the quality and quantity of intracellular lipids [43]. It is known that saturated FAs have higher melting points compared to unsaturated ones, which are often liquid at body temperature. Therefore, omega-3 FAs may potentially lower the melting point of meibomian lipids, thereby increasing their fluidity and secretion [44].

One of the earliest clinical evidence of the role of omega-3 FAs for the health of the ocular surface came from a large cross-sectional study involving over 30,000 women, which demonstrated the relationship between low dietary intake of omega-3 FAs and increased risk of DED [45]. Since then, various randomized clinical trials have demonstrated the efficacy of omega-3 FAs supplementation in DED (Table 1) [46,47,48,49,50,51,52,53,54,55,56,57,58,59,60,61]. However, a recent multicenter double-blind clinical trial (the Dry Eye Assessment and Management [DREAM] study) reported similar clinical outcomes in terms of both signs and symptoms for DED patients who received a daily oral dose of omega-3 EPA and DHA (treatment group) or an olive oil (placebo group) [62]. The “real-world” design of the study, which allowed patients to continue their DED therapy unchanged, along with the olive oil used as placebo, might have hampered the statistical significance between the two groups. It should be noted that the randomized controlled trials had significant differences regarding the doses and sources of omega-3 FAs, both of which may have an effect on their efficacy. For instance, while fish oil is rich in EPA and DHA, flaxseed oil contains short chain ALA. This might explain why the study of Macsai et al. that used the latter product did not yield significant results [57]. However, the therapeutic utility of omega-3 FAs supplementation was further confirmed by two recent meta-analyses of randomized controlled trials, which concluded that omega-3 FAs are effective in improving DED signs and symptoms [63,64].

The use of topical eye drops containing polyunsaturated FAs is currently under investigation. A previous study assessing the efficacy of topical ALA in a mice model of DED documented a positive effect as objectified by decreases in corneal CD11b+ cells, IL-1α, TNF-α expression, and conjunctival IL-1α, TNF-α, IFN γ, IL-2, IL-6 and IL-10 [65]. Furthermore, topical administration of linoleic acid have shown to enhance the stability and spreading of the lipid layer of the tear film by increasing its elasticity and compressibility [66]. A recent randomized controlled trial demonstrated a higher efficacy of a tear substitute containing flaxseed oil and trehalose compared to an analogue tear substitute without these two ingredients in ameliorating DED signs and symptoms [67].

## 3. Vitamin A

The term vitamin A comprises retinol, the most biologically active form obtained from animal sources, and carotenoids, precursors that are found in a wide variety of fruits and vegetables. Vitamin A is necessary for the health of mucosal tissues, retinal phototransduction, bone metabolism, reproduction and immune health. In particular, vitamin A is involved in the metabolism, growth and differentiation of the ocular surface epithelium [68,69].

**Figure 2:**
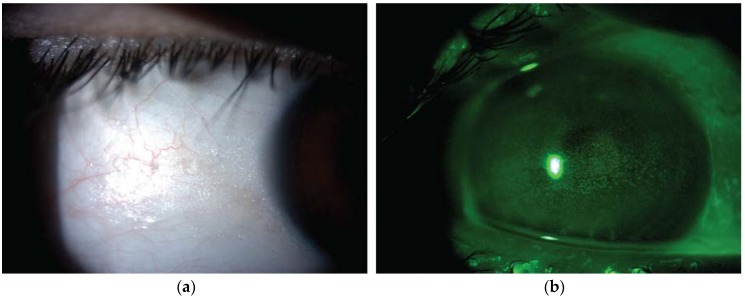
Representative image from a patient with xerophthalmia due to vitamin A deficiency secondary to bariatric surgery. (**a**) Slit lamp photograph showing dry lusterless appearance of the keratinized bulbar conjunctiva (Bitot spot). (**b**) Corneal fluorescein staining showing multiple punctate epithelial erosions within the palpebral fissure.

Vitamin A deficiency due to malnutrition is one of the major causes of preventable blindness in the developing world [70]. In Western countries, vitamin A deficiency is uncommon, and may be associated with malabsorption conditions including alcoholism, cystic fibrosis and bariatric surgery [71]. Nyctalopia represents the earliest clinical manifestations of vitamin A deficiency. In early stages of deficiency, the visual function usually normalizes following the introduction of supplementation therapy [72]. However, patients with long-term deficiency may develop ocular surface complications such as conjunctival keratinization, corneal epitheliopathy and ulceration (Figure 2a,b) [73,74].

The noticeable improvement of ocular surface epithelial damage associated either with oral or topical all-trans retinoic acid has been already previously reported for various ocular surface diseases such as DED, Stevens–Johnson syndrome, drug-induced pseudo-pemphigoid and superior limbic keratoconjunctivitis [75,76]. As demonstrated by Zhang and co-authors in a mice model of DED, vitamin A leads to the suppression of the pro-apoptotic pathway at a protein and mRNA level [77]. This is associated with a reversal of the process of squamous metaplasia, as documented by conjunctival impression cytology [75]. Furthermore, a recent clinical study has shown that short-term vitamin A supplementation in patients with DED improves the quality of tears [78].

## 4. Vitamin B12

Vitamin B12 is a cofactor in DNA synthesis and is involved in the FA and amino acid metabolism. It is found in animal products including meat, milk, egg, fish and shellfish, and its deficiency is common in patients following a vegan diet [79]. This micronutrient has a fundamental role in the synthesis of myelin, and its deficit is associated with myelopathy, peripheral neuropathy, neuropsychiatric syndromes and optic atrophy [80]. Vitamin B12 has been used in neuropathic pain associated with diabetic neuropathy, trigeminal and post-herpetic neuralgia [81].

In the last years, the role of neurosensory abnormalities in the pathophysiology of DED has become increasingly recognized. In particular, neuropathic pain due to peripheral nerve damage and/or central sensitization seems to be a common feature of the disease, which may explain the poor correlation between signs and symptoms observed in many DED patients [82]. Two recent studies showed improvement in dry eye symptoms in patients with severe dry eye disease combined or not with neuropathic ocular pain following vitamin B12 supplementation under the form of eye drops or intramuscular injection, respectively [83]. This suggests that vitamin B12 deficiency may have a role in the neurosensory abnormalities of DED.

## 5. Vitamin C

Vitamin C is a water-soluble vitamin that is required for the functioning of a wide array of enzymes and is found in fruits and vegetables like citrus fruits, strawberries, cherries, tomatoes and broccoli. The tear film contains high levels of vitamin C, reflecting the high demand of the ocular surface for antioxidant defense [84]. Furthermore, vitamin C seems to play an important role in the processes of corneal wound healing [85].

In diabetic patients, Vitamin C and E supplementation was effective in improving tear production and stability as well as goblet cell density and grade of squamous metaplasia. This was associated with a significant decrease of nitric oxide levels, which may reflect the effect of these compounds in decreasing the ocular surface oxidative stress [86]. A randomized controlled trial investigated the efficacy of an antioxidant supplementation consisting of anthocyanosides, astaxanthin, vitamins A, C, E and several herbal extracts, such *Cassiae semen* and *Ophiopogonis japonicas* in the treatment of patients with DED. After therapy, objective parameters including reactive oxygen species tear levels, Schirmer test, tear break-up time and corneal fluorescein staining significantly improved, confirming the efficacy of such preparation in enhancing lacrimal gland function, tear film stability and decreasing epithelial damage [87].

## 6. Vitamin D

Vitamin D is a fat-soluble vitamin that may be acquired via specific food intake or produced in the skin following exposure to sunlight. Vitamin D deficiency has recently been associated with the pathogenesis of DED [88]. Indeed, vitamin D plays an immunomodulatory role by suppressing the responses of both Th1 and Th2 lymphocytes. Furthermore, it regulates cellular proliferation, differentiation and apoptosis, thus enhancing corneal epithelial barrier functions. By promoting the production of surfactants, it incorporates the lipid component of tear film and tear substitutes, thus stabilizing the ocular surface system. Finally, it modulates systemic calcium absorption, which has a crucial role in maintaining a fluid secretion in both salivary and lacrimal glands [87]. Serum levels of vitamin D have shown significant correlations with tear production, stability and DE symptoms [89,90].

Starting from these assumptions, vitamin D has been investigated as a potential therapy for DED. Bae and co-authors evaluated the efficacy of vitamin D supplementation in patients with DED refractory to conventional treatment who presented vitamin D deficiency, and reported a significant improvement of Schirmer test, break-up time, corneal staining, eyelid margin hyperemia and subjective discomfort symptoms [91]. In agreement with these results, Yang and co-workers observed a significant improvement of dry eye symptoms and corneal staining after 2 months of vitamin D supplementation [92] Furthermore, the therapeutic effect of tear substitutes depends on serum vitamin D levels, and oral supplementation has shown to synergistically enhance their efficacy [93].

## 7. Selenium and Lactoferrin

Selenium is an essential micronutrient that is incorporated in a small cluster of proteins, playing a critical role in living organisms. The main dietary sources of selenium are meat, fish, seafood and cereals. The human genome contains 25 selenoprotein genes, and selenoproteins are fundamental for embryological development and human metabolism. Among these, thioredoxin reductases, glutathione peroxidases and iodothyronine deiodinases serve as oxidoreductases and depend on selenium availability for proper activity [94]. In particular, glutathione peroxidases protect cells from oxidative stress by catalyzing the reduction of hydrogen peroxide lipid hydroperoxides. This selenoprotein is widely distributed in several tissues, including the ocular surface, and its expression is reduced in patients with DED, possibly contributing to the ocular surface oxidative injury [95]. Selenoprotein P is a selenium-transport protein that is produced by the lacrimal gland and secreted in tears to provide selenium to the corneal epithelium. In DED, the levels of selenoprotein P in tears are reduced, and the shortage of selenium is thought to lead to increased oxidative stress [96]. Lactoferrin is an iron-binding glycoprotein found in most exocrine fluids including tears, which protects corneal epithelium from ultraviolet irradiation. Lactoferrin concentration was found to be reduced in DED, and oral supplementation in patients with DED, secondary to Sjögren’s syndrome, led to significant improvement of symptoms [97]. Higuchi and co-authors investigated the efficacy in dry eye animal models of selenium-binding lactoferrin, a type of lactoferrin that binds selenium instead of iron. Selenium-binding lactoferrin was incorporated into corneal epithelial cells, thus reducing oxidative damage [98]. This study indicated that selenium is a potential candidate for the clinical application in DED, as it helps restore the balance between reactive oxygen species and anti-oxidant scavengers by supporting glutathione peroxidases synthesis and function in the cornea.

## 8. Curcumin

Curcumin is a polyphenol isolated from *Curcuma longa*, which is broadly used as a spice and flavoring agent. As recent extensive research suggests, curcumin modulates multiple cell signaling pathways with resultant anti-inflammatory, antioxidant, anti-angiogenetic, wound healing and antimicrobial activities [99].

Curcumin may exert pleiotropic effects on the ocular surface system. Indeed, it down-regulates the pro-angiogenic pathway by inhibiting basic fibroblast growth factor and vascular endothelial growth factor-induced proliferation of primary endothelial cells, and thus corneal neovascularization owing to hypoxia and inflammation [100]. Moreover, curcumin-mediated wound healing promotion has suggested its potential application in conditions characterized by an impaired recovery of epithelial integrity, such as neurotrophic keratitis. In this regard, Guo and co-authors demonstrated the efficacy on intranasal delivered curcumin nanomicelle in a mice model of diabetic keratopathy. Curcumin contributed to restore ocular surface homeostasis by reducing reactive oxygen species, decreasing the expression of inflammatory mediators and increasing neurotrophic factors [101].

An additional potential application field of curcumin is DED, as notably suggested by curcumin-induced down-regulation of pro-inflammatory cytokines such as IL-4 and IL-5 in the conjunctiva of mice [102]. In human corneal epithelial cells, curcumin counteracted the increased production of IL-1β, IL-6 and TNF-α induced by hyperosmotic stress [103]. The result of this study indicates curcumin as a promising candidate for the treatment of DED.

## 9. Flavonoids

Flavonoids are a large group of polyphenols found in a variety of fruit and vegetables, tea, cocoa products and red wine. In vitro and in vivo studies have demonstrated that flavonoids have strong anti-inflammatory, immunomodulatory and antioxidative properties [104]. Quercetin is one of the most abundant and well-studied flavonoid. In an experimental mouse model of dry eye, topical application of quercetin resulted in increased tear volume, corneal regularity and goblet cell density. This was associated with a reduction of inflammatory markers, including MMP-2, MMP-9, ICAM-1 and VCAM-1 in the lacrimal gland [105]. In another dry eye mouse model, quercetin improved corneal staining and IL-1α tear concentration [106].

Green tea catechins are a group of flavonoids present in the leaves of *Camellia sinensis* that show antioxidant and anti-inflammatory activity both in vitro and in vivo [107,108,109]. The main green tea catechins are epicatechin, epigallocatechin, epicatechin gallate and epigallocatechin gallate. In a mouse model of Sjögren’s syndrome, oral administration of epigallocatechin gallate reduced the lymphocyte infiltration in the submandibular glands and protected the acinar cells from the TNF-α mediated cytotoxicity [107]. In human corneal epithelial cells, epigallocatechin gallate dose-dependently inhibited multiple cytokines induced by IL-1β or hyperosmolarity [108]. Furthermore, treatment with epigallocatechin gallate in a murine model of DED was associated with a reduction in corneal epithelial damage, number of CD11b+ cells and expression of IL-1β [109]. Masmali and co-authors evaluated the effect of a single dose of green tea on the quality and quantity of tears in healthy subjects. Surprisingly, the phenol red thread test and tear ferning tests significantly worsened following green tea consumption. Thus, the authors concluded that the high catechins content in green tea could negatively affect the tear film quality [110]. A randomized controlled trial evaluated the efficacy of green tea extract in 60 patients with DED secondary to meibomian gland dysfunction. The improvement of symptoms, break-up time and meibum quality was significantly higher in the green tea group compared to the control group [111].

Anthocyanins are a group of pigments responsible for the red, blue and purple pigmentation of many plants and fruits. The efficacy of an anthocyanin-rich extract of bilberry (*Vaccinium myrtillus* L.) was evaluated in patients with DED in a randomized controlled trial. Schirmer tests values significantly improved in patients treated with the bilberry extract, but not in control subjects. However, the study did not evaluate other important DED clinical features, such as subjective symptoms, tear film stability and epithelial damage [112]. Another study investigated the effects of maqui berry (*Aristotelia chilensis*) extract in patients with moderate DED, reporting a significant improvement of Schirmer test and quality of life after 60 days of treatments [113]. Similar results were obtained in a randomized controlled trial, which reported a significant improvement in the Schirmer test and symptoms of eye fatigue in visual display terminal users following treatment with maqui berry extract [114].

## 10. Discussion

Dry eye is an umbrella term covering a spectrum of ocular surface conditions with different underlying etiologies. Mild to moderate DED has a high prevalence and represents the most frequent complaint in the ophthalmological practice [3]. On the other hand, severe DED is often secondary to immune conditions such as Sjögren’s syndrome, rheumatoid arthritis or ocular graft-versus-host disease. These forms of DED are less common but are frequently associated with serious vision-threatening complications [115].

Novel therapeutic strategies able to address the pathophysiological mechanisms underlying DED rather than provide only symptomatic and temporary relief are currently being explored. There is growing evidence that dietary modifications and nutritional supplements may be adopted to prevent and/or treat ocular surface conditions. Micronutrients deriving from either food intake or nutraceutical products may influence the morphology and function of ocular surface components through several metabolic pathways. 

Although the use of omega-3 FAs supplementation in patients with DED is the subject of ongoing debate [116], current data available about their clinical efficacy have a convincing level of evidence [63,64]. However, the metabolic cellular pathways of omega-3 FAs as well as their molecular targets on the ocular surface need to be more deeply investigated. Furthermore, there is a need for more research to define the optimal dosage, composition (in particular in term of EPA/DHA ratio) and duration of treatment, and to identify the subgroups of patients who would potentially benefit more from this treatment.

Vitamin A deficiency is one the most prevalent micronutrient deficiencies worldwide and may lead to xerophthalmia, which represents the leading cause of preventable blindness in the developing world [70]. Vitamin C and D play important roles in ocular surface homeostasis, while vitamin B12 might be involved in DED with and without neuropathic pain [83,117]. In patients with ocular surface disorders, vitamin deficiencies should be promptly identified and corrected. Nevertheless, the efficacy of oral vitamin supplementations in the general DED population is still unproven. Therefore, their use cannot be recommended at this time in all patients with DED.

There is a great interest in the possible protective effect of polyphenols and flavonoids on the ocular surface. The promising results of in vitro and animal models identify these molecules as potential candidates for clinical application in DED [102,103,105,106,107,108,109]. However, their poor bioavailability due to limited absorption and rapid elimination may limit their clinical efficacy [118]. In addition, high doses of some of these compounds could exhibit undesired effects on the tear film quality [110]. Therefore, large, appropriately controlled clinical trials are still required to provide conclusive evidence regarding the efficacy of polyphenols and flavonoids on ocular surface disorders.

The majority of the available studies on nutritional supplementation for DED did not evaluate the micronutrients dietary intake nor their plasma level, and this represents the major limitation of the existing literature. Background diet should be considered a confounder in supplementation trials [119], and different dietary practices might explain the heterogeneous results of studies conducted in different world regions. For instance, a recent meta-analysis reported a higher efficacy of omega-3 FAs supplementation in studies conducted in India, where diet is principally vegetarian, with negligible intakes of long-chain omega-3 FAs [63]. Another limitation is that most of the clinical trials had a short follow-up. Thus, it is still unknown if the reported results are maintained over time. Finally, almost all the studies evaluated the effect of a single supplement. Therefore, the possible synergistic effect of a combination of different micronutrients and nutraceuticals still needs to be investigated.

Despite the promising results of the nutritional supplements for DED, the high cost of most products represents a major drawback for their use. This may lead to decreased treatment adherence, particularly in those patients who are on polytherapy. Indeed, high number of tablets and cost are common reasons for poor adherence to nutritional supplementation [120]. Finally, cost-effectiveness evaluations are still required to support the clinical use of nutritional supplements in patients with ocular surface disorders.

## 11. Conclusions

In conclusion, a large number of dietary compounds seems to have positive effects on the ocular surface health. In particular, omega-3 FAs have the strongest level of evidence in support of their efficacy in DED, but optimal dosage, formulation and duration of treatment still need to be defined. Further clinical research is advisable to confirm the safety and efficacy of the supplementation with the other micronutrients and nutraceuticals.

## Figures and Tables

**Table 1:** Characteristics of high level of evidence studies (randomized controlled trials and meta-analyses) evaluating the efficacy of omega-3 fatty acids in dry eye disease.

Author (Year)	Etiology of Dry Eye	No. of Patients	Omega-3 FAs Daily Dose	Outcome
Asbell et al. (2018)	Not specified	535	EPA 2000 mg + DHA 1000 mg	No differences in OSDI corneal and conjunctival staining, TBUT, and Schirmer test compared to placebo
Bhargava et al. (2013)	Not specified	518	EPA 650 mg + DHA 350 mg	Significant improvement in symptom score, Schirmer test and TBUT compared to placebo
Bhargava et al. (2015)	Visual display terminal users	456	EPA 360 mg + DHA 240 mg	Significant improvement in symptom score, Schirmer test and TBUT, Nelson grade and goblet cell density compared to placebo
Bhargava et al (2015b)	Contact lens	496	EPA 720 mg + DHA 480 mg	Significant improvement in symptom score, lens wear comfort level, TBUT and Nelson grade compared to placebo
Bhargava et al. (2016)	Visual display terminal users	522	EPA 1440 mg + DHA 960 mg	Significant improvement in symptom score, TBUT and Nelson grade compared to placebo
Bhargava et al. (2016b)	Rosacea	130	EPA 720 mg + DHA 480 mg	Significant improvement in symptom score, TBUT and Schirmer test and Meibomian gland score compared to placebo
Brignole-Baudouin et al. (2011)	Sjögren and non-Sjögren	121	EPA 427.5 mg + DHA 285 mg + borage oil 15 mg	Significant reduction in the percentage of HLA-DR-positive conjunctival cells. No difference in signs and symptoms
Deiniema et al. (2017)	Not specified	54	Krill oil (EPA 945 mg + DHA 510 mg) and Fish oil (EPA 1000 mg + DHA 500 mg)	Significant improvement in OSDI, tear osmolarity, TBUT, bulbar redness and IL-17 levels compared to placebo
Epitropoulos et al. (2016)	MGD	105	EPA 1680 mg + DHA 560 mg	Significant improvement in OSDI, tear osmolarity, TBUT, and MMP-9 positivity compared to placebo.
Kangari et al. (2013)	Not specified	64	EPA 360 mg + DHA 240 mg	Significant improvement in OSDI, TBUT, and Schirmer test compared to placebo
Kawakita et al. (2013)	Not specified	26	EPA 1245 mg + DHA 540 mg	Significant improvement of eye pain, TBUT and rose bengal staining compared to placebo
Larmo et al. (2010)	Not specified	100	Sea buckthorn oil (2 g): long chain omega-3 FAs 149 mg + omega-6 FAs 245 mg	Significant improvement of bulbar redness and tear osmolarity compared to placebo
Macsai et al. (2008)	MGD	38	Flaxseed oil 6000 mg	No significant difference in OSDI, meibum score, TBUT compared to placebo
Malhotra et al. (2015)	MGD	60	EPA 720 mg + DHA 480 mg	Significant improvement of OSDI, tear break-up time, ocular surface staining, meibum quality and expressibility and contrast sensitivity compared to placebo
Oleñik et al. (2013)	MGD	64	EPA 127.5 mg + DHA 1050 mg	Significant improvement in OSDI, TBUT, lid margin inflammation and meibum expressibility compared to placebo
Sheppard et al. (2013)	Not specified	38	ALA 196 mg + EPA 128 mg + DHA 99 mg + DPA 39 mg + LA 710 mg + GLA 240 mg	Significant improvement in OSDI, surface asymmetry index and HLA-DR expression compared to placebo
Wojtowicz et al. (2011)	Not specified	36	EPA 450 mg + DHA 300 mg + flaxseed oil 100 mg	No significant difference in Schirmer testing, fluorophotometry and composition of meibomian gland secretion sample compared to control group
Chi et al. (2019) *	Nonspecific dry eye disease	1782	Different doses	Significant improvement in OSDI, TBUT, Schirmer test and tear osmolarity compared to placebo
Giannaccare et al. (2019) *	Different etiologies	3363	Different doses	Significant improvement in dry eye symptoms, TBUT, Schirmer test and corneal staining

ALA: alpha-linolenic acid; DHA: docosahexaenoic acid; DPA: docosapentaenoic acid; EPA: eicosapentaenoic acid; FAs: fatty acids; GLA: gamma-linolenic acid; IL: intereukin; LA: linolenic acid; MGD: meibomian gland dysfunction; MMP: matrix metalloproteinases; OSDI: ocular surface disease index; TBUT: tear film break-up time. * Meta-analysis.
